# Oxygen Storage Capacity and CO Oxidation Performance of CeO_2_ Nano-Octahedra with Saturated In^3+^ Doping

**DOI:** 10.3390/nano16080474

**Published:** 2026-04-17

**Authors:** Chang Chen, Yaohui Xu, Qin Wang, Zhao Ding

**Affiliations:** 1 Laboratory for Functional Materials, School of New Energy Materials and Chemistry, Leshan Normal University, Leshan 614000, China; chenchang@lsnu.edu.cn (C.C.); wq306115@lsnu.edu.cn (Q.W.); 2 Leshan West Silicon Materials Photovoltaic and New Energy Industry Technology Research Institute, Leshan 614000, China; 3 National Engineering Research Center for Magnesium Alloys, College of Materials Science and Engineering, Chongqing University, Chongqing 400044, China

**Keywords:** CeO_2_, In^3+^ doping, oxygen storage capacity, CO oxidation, nano-octahedra, surface hydroxyls

## Abstract

CeO_2_ is widely studied in catalysis owing to its Ce^4+^/Ce^3+^ redox couple and oxygen storage capacity (OSC), but its low-temperature redox activity remains a challenge. To address this, this study investigates the effects of saturated In^3+^ doping (1 mol.%) on the structural, redox, and catalytic properties of nano-octahedral CeO_2_. Structural and chemical analyses reveal that In^3+^ doping induces lattice contraction from 5.4171 to 5.4129 Å, increases oxygen vacancy concentration from 29.7% to 39.8%, and raises surface Ce^3+^ fraction from 27.6% to 30.0%. Consequently, H_2_-TPR measurements show that the surface reduction peak temperature decreases from 548 to 406 °C and the onset reduction temperature shifts from 309 °C to 183 °C. Quantitative OSC analysis further demonstrates that the low-temperature OSC increases from 13.17 to 20.57 mmol O_2_/mol and the high-temperature OSC from 53.36 to 59.38 mmol O_2_/mol upon doping. As a result of these enhancements, CO-TPSR tests reveal improved low-temperature CO oxidation performance, with the CO_2_ light-off temperature decreasing from 99 to 72 °C and the rapid oxidation temperature from 153 to 96 °C. Notably, H_2_O and H_2_ signals are detected during CO-TPSR, and FTIR analysis confirms the enrichment of surface hydroxyl groups in the doped sample, offering new mechanistic insights into the involvement of surface species in the reaction pathway. Overall, saturated In^3+^ doping effectively enhances the oxygen vacancy concentration, surface reducibility, and CO oxidation activity of nano-octahedral CeO_2_.

## 1. Introduction

Cerium dioxide (CeO_2_), as a key rare-earth functional material, has been widely employed in various fields such as automotive exhaust purification (e.g., three-way catalysts), solid oxide fuel cells, water-gas shift reactions, and CO oxidation, owing to its unique Ce^4+^/Ce^3+^ redox couple and excellent OSC [1,2,3]. The OSC of CeO_2_ originates from a reversible redox cycle: under oxygen-deficient conditions, Ce^4+^ can be reduced to Ce^3+^ with the release of lattice oxygen, while under oxygen-rich conditions, Ce^3+^ can be re-oxidized to Ce^4+^ accompanied by the uptake of gaseous oxygen [4,5,6]. This characteristic enables CeO_2_ to dynamically regulate the release and capture of oxygen species. However, although CeO_2_ exhibits superior redox activity at elevated temperatures, its activity under low-temperature conditions (e.g., during the cold-start period of automobiles) remains relatively limited, which significantly constrains its practical performance. Consequently, enhancing the OSC and redox activity of CeO_2_ at low temperatures has long been a research focus in this field [7].

Ion doping has proven to be an effective strategy for improving the OSC of CeO_2_ [8,9]. From the perspective of crystal chemistry, the structural stability of CeO_2_ in the cubic fluorite phase is closely related to the ratio of the cation radius to the anion radius (*r*^+^/*r*^−^). According to the radius ratio rule, a stable eight-coordinated cubic ionic crystal requires *r*^+^/*r*^−^ ≥ 0.732. In CeO_2_, however, the radius ratio of Ce^4+^ (0.97 Å) to O^2−^ (1.38 Å) is approximately 0.703, slightly below the theoretical stability threshold [10]. To maintain structural stability, a fraction of Ce^4+^ in CeO_2_ spontaneously reduces to Ce^3+^, which possesses a larger ionic radius, a phenomenon known as “cerium self-doping”, accompanied by the formation of oxygen vacancies (*V*_O_) [11]. This mechanism not only serves as the primary source of intrinsic *V*_O_ in CeO_2_ but also reveals the profound influence of ionic radius on crystal stability and defect chemistry.

Building on this understanding, the selection of appropriate foreign dopant ions can further modulate the defect concentration and redox properties of CeO_2_ [12,13]. The choice of dopant ions is primarily guided by two factors. On the one hand, the size effect means that incorporating ions with a radius smaller than that of Ce^4+^ can induce lattice contraction and affect the formation energy of *V*_O_ [14]. On the other hand, the charge compensation effect requires that introducing ions with a valence lower than +4 forces the system to generate additional *V*_O_ to maintain electrical neutrality [15]. In^3+^ satisfies both criteria: its effective ionic radius (0.92 Å for eight-coordination) is smaller than that of Ce^4+^ (0.97 Å), and its valence state is +3 [16]. Unlike isovalent ions such as Zr^4+^, the heterovalent doping of In^3+^ allows for direct charge compensation via oxygen vacancies, thereby enhancing the *V*_O_ concentration more efficiently [17,18]. Furthermore, the electronegativity of In^3+^ is higher than that of Ce^4+^, and the relatively strong In-O bond may modulate the local electronic structure of lattice oxygen, consequently influencing the reactivity of oxygen species [19]. In our previous work, we systematically investigated the solid solubility behavior of In^3+^ in CeO_2_ and established that the saturated solid solubility of In^3+^ in CeO_2_ lattice is approximately 1 mol.% [20]. Exceeding this concentration leads to the precipitation of a secondary In_2_O_3_-like phase, which in turn results in decreased specific surface area, reduced *V*_O_ concentration, and diminished OSC. Therefore, the saturated doping concentration represents an optimal balance, maximizing the beneficial effects of In^3+^ incorporation while avoiding the deleterious effects of phase segregation. This finding provides the rationale for selecting 1 mol.% as the doping concentration in the present study, ensuring that In^3+^ is homogeneously distributed within the CeO_2_ lattice in the form of a solid solution.

In addition to doping, the catalytic performance of CeO_2_ exhibits a pronounced morphology dependence [21,22]. CeO_2_ samples with different morphologies expose distinct crystal facets, which differ in surface energy, *V*_O_ formation energy, and reactant adsorption capacity, thereby affecting their redox activity and catalytic properties [23,24]. Notably, the nano-octahedral morphology predominantly exposes the thermodynamically stable (111) facet, which possesses the lowest surface energy and exhibits favorable catalytic activity and thermal stability in reactions such as CO oxidation [25]. In previous work, we developed a synthesis method independent of traditional organic templates, achieving the controllable synthesis of CeO_2_ with diverse morphologies, including nanosheets, dendrites, octahedra, and hollow structures, by regulating the amount of NH_4_HCO_3_ and the reaction time in a methanol-water mixed solvent system [26]. Among these, the nano-octahedra exhibit uniform size (approximately 240 nm), good dispersion, and predominantly expose the (111) facet, making them suitable as a matrix material for doping modification to investigate doping effects on specific crystal facets.

Guided by these insights, this study focuses on 1 mol.% In^3+^-doped nano-octahedral CeO_2_, the concentration corresponding to the saturated solid solubility established in our previous work, as a model system to investigate how aliovalent doping modulates the structural and functional properties of ceria. A multi-technique approach comprising XRD, XPS, H_2_-TPR, and CO-TPSR is employed to establish correlations between In^3+^ incorporation, defect chemistry (*V*_O_ concentration and Ce^3+^ fraction), reducibility, and CO oxidation activity. Notably, signals corresponding to H_2_O and H_2_ were observed during the CO-TPSR measurements, which may offer new insights into the involvement of surface species in the oxidation pathway. By integrating structural, chemical, and catalytic analyses, this work aims to elucidate the mechanism by which saturated In^3+^ doping enhances the low-temperature performance of nano-octahedral CeO_2_, building upon our previous investigations into solid solubility and morphology-controlled synthesis.

## 2. Materials and Methods

### 2.1. Starting Materials

Cerium(III) nitrate hexahydrate (Ce(NO_3_)_3_·6H_2_O, 99.95%) and ammonium bicarbonate (NH_4_HCO_3_, 99.995%) were purchased from Aladdin Co., Ltd. (Shanghai, China). Indium(III) nitrate (In(NO_3_)_3_, 99.99%) was purchased from Shanghai ACMEC Biochemical Technology Co., Ltd. (Shanghai, China). Methanol (99.5%) was obtained from Chengdu Kelong Chemical Co., Ltd. (Chengdu, China). Purified water, which was used for preparing aqueous stock solutions and washing the products throughout all experiments.

### 2.2. Synthesis of 1 mol.% In-Doped CeO_2_ and Pure CeO_2_

**Figure 1:**
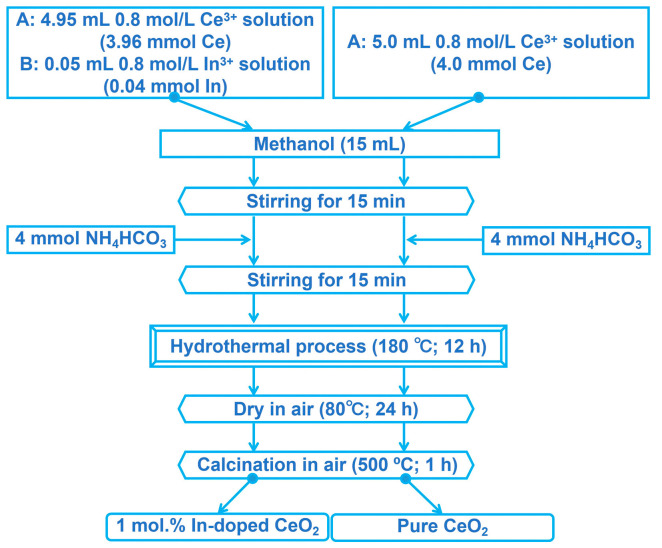
Schematic illustration of the synthesis procedure for pure CeO_2_ and 1 mol.% In-doped CeO_2_ samples via a hydrothermal method (180 °C, 12 h) followed by calcination (500 °C, 1 h).

The doping concentration of 1 mol.% was selected based on our previous determination of the saturated solid solubility of In^3+^ in CeO_2_ (approximately 1 mol.%), which represents the maximum concentration achievable without secondary phase precipitation. The 1 mol.% In-doped and pure CeO_2_ samples were synthesized via a combined hydrothermal and calcination method, as illustrated in Figure 1. The detailed steps are as follows.

First, aqueous stock solutions of Ce(NO_3_)_3_·6H_2_O and In(NO_3_)_3_ were prepared, each at a concentration of 0.8 M (mol/L). For the 1 mol.% In-doped CeO_2_ sample, 4.95 mL of the Ce^3+^ stock solution (containing 3.96 mmol of Ce, denoted as Solution A) was precisely mixed with 0.05 mL of the In^3+^ stock solution (containing 0.04 mmol of In, denoted as Solution B) using a micropipette. For the pure CeO_2_ sample, 5.0 mL of the Ce^3+^ stock solution (containing 4.0 mmol of Ce) was accurately transferred.

Subsequently, 15 mL of methanol was added to each 5.0 mL aqueous mixture, followed by magnetic stirring for 15 min. Then, 4 mmol of NH_4_HCO_3_ was introduced into each mixture, with stirring continued for an additional 15 min.

The resulting mixtures were separately transferred into Teflon-lined stainless-steel autoclaves. They were placed in a forced-air drying oven (LC-101-2B, LICHEN, Changsha, China), and the oven was then heated from room temperature to 180 °C over approximately 30 min, corresponding to an estimated average heating rate of about 5 °C/min, and maintained at 180 °C for 12 h. After the reaction, the autoclaves were allowed to cool naturally to room temperature. The products were collected, thoroughly washed with purified water, and dried in air at 80 °C for 24 h. Finally, the dried precursors were placed in a muffle furnace (SX2-5-12, Jindian Instrument Co., Ltd., Yuyao, China). The furnace was heated from room temperature to 500 °C over approximately 30 min, corresponding to an average heating rate of about 15 °C/min. The calcination was then carried out in air at 500 °C for 1 h, after which the furnace was turned off and the sample was allowed to cool naturally to room temperature to obtain the final powder samples.

### 2.3. Characterization

The crystalline phases of the samples were identified by X-ray diffraction (XRD) using a DX-2700 diffractometer (Dandong Haoyuan Instrument Co., Ltd., Dandong, China) with Cu Kα radiation (λ = 1.5406 Å), operated at 30 kV and 25 mA. Patterns were recorded from 20° to 80° with a step size of 0.05°. Crystallite size, lattice strain, relative crystallinity, and lattice parameters were calculated from the (111) diffraction peak (2θ ≈ 28.54°) using MDI Jade 6.0. Specifically, the crystallite size was determined using the Scherrer equation, the lattice strain was calculated from the peak broadening, and the relative crystallinity was obtained from the integrated intensity ratio of the (111) peak. These values represent a relative comparison between samples rather than absolute crystallinity percentages. Morphology and elemental distribution were examined by scanning electron microscopy (SEM, SEM5000, CIQTEK, Hefei, China) at 3.0 kV, equipped with energy-dispersive X-ray spectroscopy (EDS). The actual doping amount of In in the 1 mol.% In-doped CeO_2_ sample was determined using inductively coupled plasma-atomic emission spectrometry (ICP-AES, SPECTRO ARCOS EOP, Kleve, Germany). High-resolution transmission electron microscopy (HRTEM) was performed on a JEM-2100F instrument (JEOL Ltd., Tokyo, Japan) to observe lattice fringes and confirm the exposed crystal facets. Specific surface areas (*S*_BET_) of the calcined samples were determined by the Brunauer-Emmett-Teller (BET) method using nitrogen physisorption on a TriStar II Plus analyzer (Micromeritics Instrument Corporation, Norcross, GA, USA). X-ray photoelectron spectroscopy (XPS) measurements were conducted on an ESCALAB 250Xi spectrometer (Thermo Fisher Scientific, Waltham, MA, USA) using monochromatic Al Kα radiation. Survey spectra were recorded with a pass energy of 100 eV (step 1 eV), and high-resolution spectra of O 1s and Ce 3d with a pass energy of 20 eV (step 0.05 eV). Binding energies were calibrated using adventitious carbon C 1s at 284.8 eV. Peak fitting was performed with XPS Peak 4.1 software.

The OSC of the pure and 1 mol.% In-doped CeO_2_ samples was evaluated by hydrogen temperature-programmed reduction (H_2_-TPR) using a TP-5080D automatic chemisorption analyzer (Tianjin Xianquan Industry and Trade Development Co., Ltd., Tianjin, China). Approximately 50 mg of sample was pretreated in 5% O_2_/N_2_ (100 mL/min) at 500 °C for 1 h, cooled to room temperature, and purged with N_2_. Reduction was performed in 5% H_2_/N_2_ (30 mL/min) from room temperature to 1020 °C at 10 °C/min, and H_2_ consumption was monitored by a thermal conductivity detector (TCD).

The catalytic oxidation performance of the CeO_2_-based samples toward CO was investigated by CO temperature-programmed surface reaction (CO-TPSR) coupled with on-line mass spectrometry (MS). About 50 mg of sample was pretreated in N_2_ (100 mL/min) at 200 °C for 1 h, cooled to room temperature, and then exposed to a flow of 1% CO + 10% O_2_ + 89% He (40 mL/min). After stabilizing the baseline, the temperature was raised to 850 °C at 10 °C/min. The effluent gases were continuously monitored by an on-line mass spectrometer, and the signals at specific mass-to-charge ratios (*m*/*z*) corresponding to CO (*m*/*z* = 28), O_2_ (*m*/*z* = 32), CO_2_ (*m*/*z* = 44), H_2_O (*m*/*z* = 18), and H_2_ (*m*/*z* = 2) were recorded.

## 3. Results and Discussion

### 3.1. XRD Analysis

**Figure 2:**
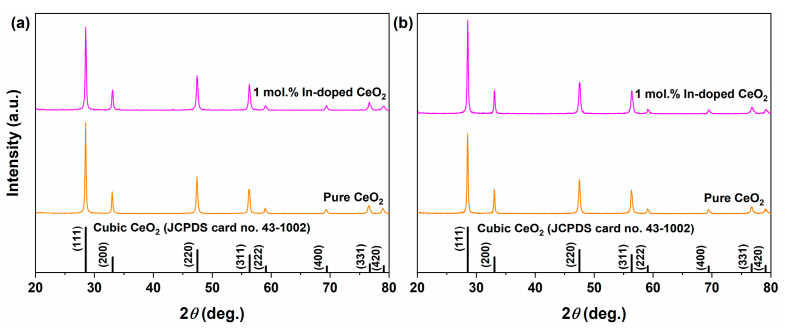
XRD patterns of pure and 1 mol.% In-doped CeO_2_ samples: (**a**) before calcination (directly after hydrothermal reaction at 180 °C for 12 h) and (**b**) after calcination in air at 500 °C for 1 h.

XRD analysis was performed on pure and 1 mol.% In-doped CeO_2_ samples before and after calcination to investigate the effects of hydrothermal synthesis and subsequent thermal treatment on phase composition and crystal structure, and to verify whether In^3+^ was successfully incorporated into the CeO_2_ lattice. Figure 2a and Figure 2b display the XRD patterns of the samples before and after calcination, respectively, and the corresponding structural parameters (crystallite size, relative crystallinity, lattice strain, and lattice parameters) are summarized in Table 1.

For the uncalcined samples, i.e., those obtained directly after the hydrothermal reaction at 180 °C for 12 h (Figure 2a), both exhibit characteristic diffraction peaks corresponding to the cubic fluorite structure (JCPDS No. 43-1002), with no detectable In_2_O_3_ or other impurity phases. This indicates that the hydrothermal method itself directly yields phase-pure CeO_2_. However, phase purity does not equate to structural perfection. For pure CeO_2_ before calcination, the crystallite size is 18.2 nm, the lattice strain is 0.481%, and the lattice parameter is 5.4278 Å. For the 1 mol.% In-doped sample before calcination, the crystallite size is slightly smaller (17.8 nm), while the lattice strain (0.540%) and the lattice contraction (5.4205 Å) are more pronounced. These differences reflect the lattice distortion induced by the substitution of Ce^4+^ by the smaller In^3+^ ion and the generation of charge-compensating *V*_O_.

For the calcined samples, i.e., those subsequently heated in air at 500 °C for 1 h (Figure 2b), the diffraction peaks become sharper and more symmetric, with narrowed full width at half maximum. This transformation reflects a thermodynamically driven process of lattice rearrangement and defect annihilation. Quantitatively (Table 1), calcination significantly relieves lattice strain, with values decreasing from 0.481% to 0.361% for pure CeO_2_ and from 0.540% to 0.349% for the doped sample. Calcination also promotes moderate crystallite growth, from 18.2 to 27.4 nm for pure CeO_2_ and from 17.8 to 30.2 nm for the doped sample, which reduces grain boundary density. In addition, the lattice parameters exhibit a regular contraction, decreasing from 5.4278 to 5.4171 Å for pure CeO_2_ and from 5.4205 to 5.4129 Å for the doped sample, indicating atomic densification. Thus, calcination elevates the hydrothermal product from phase purity to structural perfection by repairing defects, relieving strain, and optimizing lattice periodicity.

Evidence for successful In^3+^ incorporation can be obtained from the structural parameters summarized in Table 1. The lattice parameter of the doped sample is consistently smaller than that of pure CeO_2_ both before and after calcination (5.4205 vs. 5.4278 Å before calcination; 5.4129 vs. 5.4171 Å after calcination). This systematic contraction arises from the substitution of Ce^4+^ (0.97 Å, eight-coordinate) by the smaller In^3+^ ion (0.92 Å). Notably, this contraction is already present before calcination, demonstrating that In^3+^ incorporation occurs during hydrothermal crystallization rather than being induced by the subsequent thermal treatment. The higher lattice strain of the doped sample before calcination (0.540% vs. 0.481%) is attributed to additional lattice distortion caused by charge-compensating *V*_O_ generated upon heterovalent substitution. After calcination, the strain in the doped sample decreases more substantially (from 0.540% to 0.349%, a 35.4% reduction) than in the pure sample (from 0.481% to 0.361%, a 25.0% reduction), indicating effective healing of extrinsic defects. The relative crystallinity of the doped sample remains consistently lower, by approximately 2.0%, than that of the pure sample both before and after calcination, consistent with the presence of residual defects. Furthermore, the significant difference in Pauling electronegativity between Ce (1.12) and In (1.78) implies stronger covalent character of In-O bonds, which helps anchor dopant ions and inhibit surface segregation during calcination, thereby maintaining a homogeneous solid solution without secondary phase formation [27]. Collectively, these multiple lines of evidence conclusively demonstrate that In^3+^ has successfully entered the CeO_2_ lattice and occupied Ce^4+^ sites.

The evolution of crystallite size and crystallinity further supports these findings. The slightly larger crystallite growth in the doped sample (from 17.8 to 30.2 nm) compared to the pure sample (from 18.2 to 27.4 nm) may be related to enhanced ion diffusion promoted by *V*_O_. This moderate growth reduces grain boundary density, which benefits oxygen transport and thus contributes to enhanced catalytic activity. The marginal increases in relative crystallinity upon calcination, from 63.2% to 64.3% for pure CeO_2_ and from 61.2% to 62.3% for the doped sample, reaffirm that the hydrothermal method provides high initial crystallinity, with calcination primarily optimizing crystal quality rather than increasing crystallinity itself. These structural findings lay a solid foundation for understanding the subsequent OSC performance, reduction behavior, and catalytic activity of the In-doped CeO_2_ samples.

### 3.2. SEM, HRTEM, BET and Elemental Distribution

**Figure 3:**
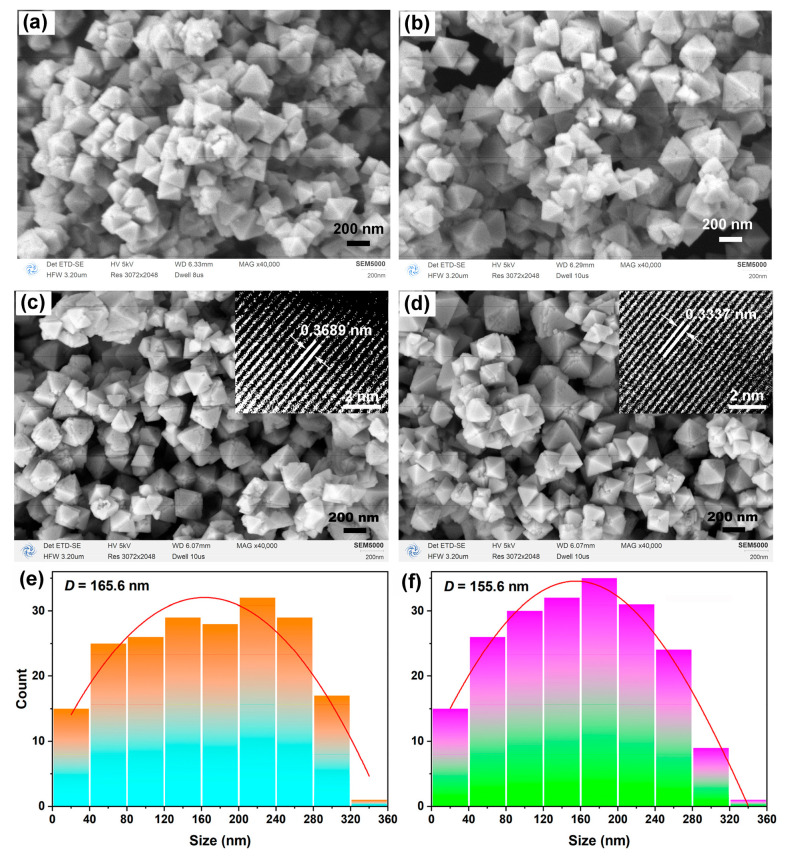
SEM images of (**a**) pure CeO_2_ and (**b**) 1 mol.% In-doped CeO_2_ before calcination; (**c**) pure CeO_2_ and (**d**) 1 mol.% In-doped CeO_2_ after calcination at 500 °C for 1 h (Insets in (**c**,**d**) are the corresponding HRTEM images); the corresponding particle size distributions determined by statistical analysis of the SEM images using ImageJ software for (**e**) pure CeO_2_ and (**f**) 1 mol.% In-doped CeO_2_.

The morphology of pure and 1 mol.% In-doped CeO_2_ before and after calcination was examined by SEM, as shown in Figure 3a–d.

Before calcination (Figure 3a,b), both samples consist of well-defined octahedral particles with smooth surfaces, similar to their calcined counterparts (Figure 3c,d), indicating that the octahedral morphology is primarily established during the hydrothermal synthesis. The main effect of subsequent calcination at 500 °C is to improve crystallinity and remove residual organic species, rather than to alter the particle shape. After calcination (Figure 3c,d), the octahedral particles remain well defined with smooth surfaces and good dispersion. From Figure 3a–d, a comparison reveals that In^3+^ doping does not change the octahedral morphology, consistent with the XRD results showing no phase transformation upon doping. Particle size distributions for the calcined samples, obtained from statistical analysis of the SEM images using ImageJ software (1.8.0), are presented in Figure 3e,f. The average equivalent circular diameter decreases slightly from 165.6 nm for pure CeO_2_ to 155.6 nm for the doped sample, a reduction of approximately 10 nm. Notably, the crystallite size calculated from XRD (Table 1) shows an opposite trend, increasing from 27.4 nm to 30.2 nm upon doping. This apparent discrepancy arises because SEM measures the overall size of individual particles, whereas XRD determines the size of coherently scattering domains (crystallites). These two parameters reflect structural information at different length scales and are not directly correlated. The increase in crystallite size may be attributed to enhanced ion diffusion promoted by *V*_O_ generated upon In^3+^ doping, while the slight decrease in particle size could be related to doping effects on the nucleation process [28]. Specifically, during the initial stage of hydrothermal crystallization, the presence of In^3+^ ions in the precursor solution can influence the nucleation kinetics of CeO_2_. The significantly higher Pauling electronegativity of In (1.78) compared to Ce (1.12) implies that In–O bonds possess stronger covalent character than Ce-O bonds. This stronger covalency favors the preferential incorporation of In^3+^ into the early-stage nuclei, as the formation of In-O bonds reduces the overall free energy of the critical nuclei. This preferential incorporation can increase the nucleation rate by lowering the critical free energy barrier for nucleation, leading to the formation of a larger number of nuclei within the same reaction volume. Under such conditions, the available solute species are distributed among more nuclei, thereby restricting the growth of individual particles and resulting in a smaller average particle size. Furthermore, the charge-compensating oxygen vacancies generated upon heterovalent substitution can alter the local coordination environment of cerium ions and affect the diffusion kinetics of solute species. This combined effect, namely accelerated nucleation coupled with possibly retarded coarsening, contributes to the slightly reduced particle size observed for the In-doped sample. The increased lattice strain in the doped sample before calcination (0.540% vs. 0.481% for pure CeO_2_, Table 1) further supports the argument that dopant incorporation induces structural distortion during the nucleation stage, which is consistent with the observed particle size reduction.

To further confirm the exposed crystal facets and the effect of In doping on the lattice spacing, HRTEM was performed on the calcined samples, with the images shown as insets in Figure 3c,d. For pure CeO_2_, the lattice fringes exhibit a measured spacing of 0.3389 nm (average of 20 measurements). For the 1 mol.% In-doped CeO_2_, the lattice spacing is determined to be 0.3137 nm (average of 20 measurements), which is in excellent agreement with the *d*-spacing of the (111) plane of cubic fluorite CeO_2_ (JCPDS No. 43-1002, 0.3124 nm). The slightly larger spacing observed for the pure sample may be attributed to residual surface strain or the presence of surface defects. The decreased lattice spacing upon doping is consistent with the substitution of Ce^4+^ (0.97 Å) by the smaller In^3+^ ion (0.92 Å) and corroborates the lattice contraction observed by XRD (from 5.4171 to 5.4129 Å). These HRTEM results directly confirm that both samples predominantly expose the (111) facet, which is known to be the most stable surface for CeO_2_ and favorable for CO oxidation [29].

The specific surface areas of the calcined samples were determined by the BET method, and the results are summarized in Table 1. The *S*_BET_ of pure CeO_2_ is 34.4 m^2^/g, while that of 1 mol.% In-doped CeO_2_ increases to 40.2 m^2^/g. The modest increase in surface area upon doping can be attributed to the slightly smaller particle size observed by SEM (155.6 nm vs. 165.6 nm) and the enhanced surface roughness due to the generation of *V*_O_.

**Figure 4:**
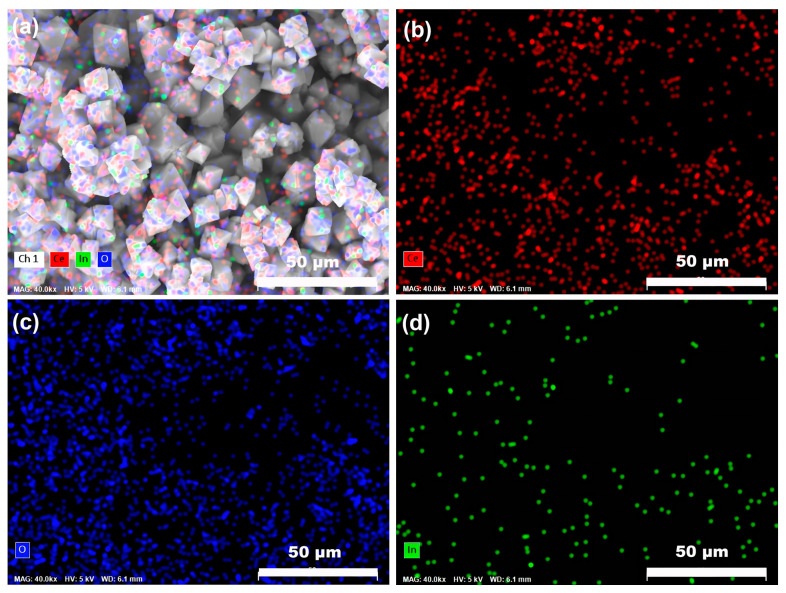
(**a**) SEM image of the selected area and corresponding EDS elemental mappings of (**b**) Ce, (**c**) O, and (**d**) In for the 1 mol.% In-doped CeO_2_ sample.

Elemental distribution within the doped sample was investigated by EDS mapping, as shown in Figure 4. The selected SEM area (Figure 4a) and corresponding elemental maps for Ce (Figure 4b), O (Figure 4c), and In (Figure 4d) reveal uniform distribution of all elements throughout the particles, with no evidence of localized enrichment or segregation. The In signal closely follows the particle contours, confirming homogeneous incorporation of In^3+^ into the CeO_2_ particles rather than surface adsorption or formation of separate oxide phases. ICP-AES analysis shows that the actual In content in the doped sample is approximately 0.94 mol.%, slightly lower than the nominal 1 mol.% due to possible In^3+^ loss during hydrothermal and washing steps. Together with the absence of In-related impurity peaks in XRD and the systematic lattice contraction discussed earlier, these results demonstrate that In^3+^ is successfully incorporated into the CeO_2_ lattice, forming a homogeneous solid solution. These morphological and compositional findings provide a reliable foundation for understanding the subsequent oxygen storage and catalytic properties of the materials.

### 3.3. XPS Analysis

**Figure 5:**
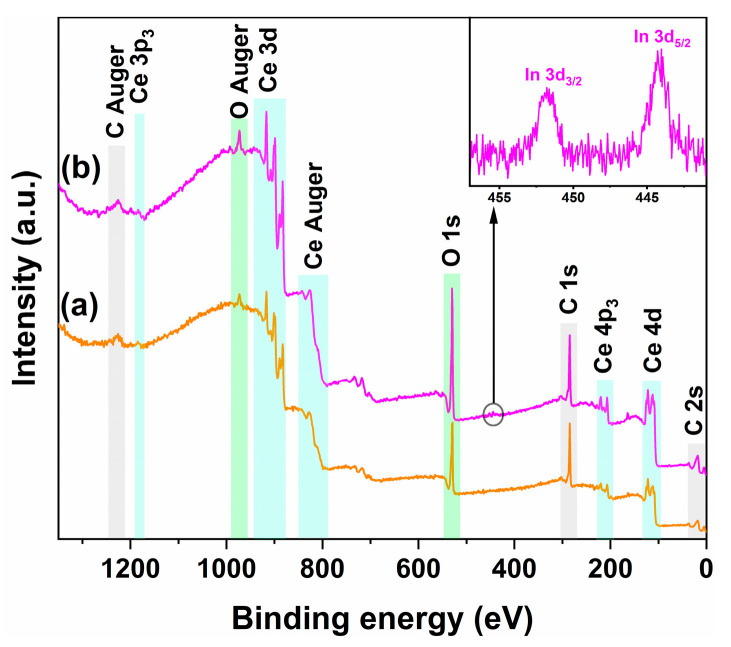
XPS survey spectra of (**a**) pure CeO_2_ and (**b**) 1 mol.% In-doped CeO_2_. (Inset is the corresponding In 3d XPS regions for the doped CeO_2_).

**Figure 6:**
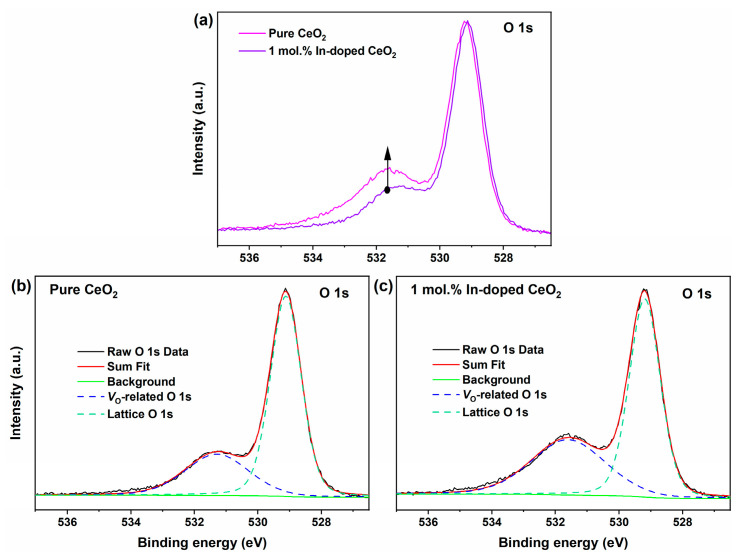
(**a**) O 1s core-level XPS spectra of pure and 1 mol.% In-doped CeO_2_; fitted curves for (**b**) pure CeO_2_ and (**c**) 1 mol.% In-doped CeO_2_.

**Figure 7:**
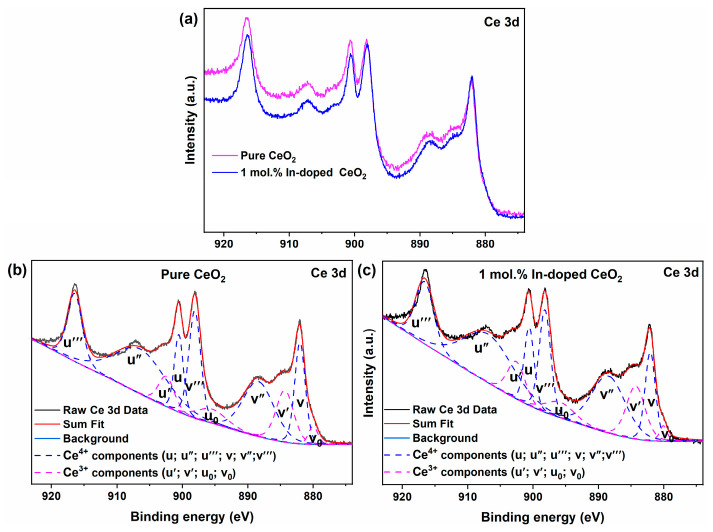
(**a**) Ce 3d core-level XPS spectra of pure and 1 mol.% In-doped CeO_2_; fitted curves for (**b**) pure CeO_2_ and (**c**) 1 mol.% In-doped CeO_2_.

To investigate the effect of In doping on the surface chemical states, *V*_O_ concentration, and cerium valence distribution of CeO_2_, XPS was performed on the calcined pure CeO_2_ and 1 mol.% In-doped CeO_2_ samples. Figure 5 displays the survey spectra and the high-resolution In 3d spectrum, while Figure 6 and Figure 7 show the core-level spectra and corresponding peak fitting results for O 1s and Ce 3d, respectively.

#### 3.3.1. Surface Elemental Composition and Confirmation of In Doping

Figure 5a and Figure 5b present the XPS survey spectra of pure CeO_2_ and 1 mol.% In-doped CeO_2_, respectively. Within the survey scan range, both samples exhibit complete characteristic signals of the CeO_2_ matrix. A weak, broad peak located at approximately 1184.1 eV corresponds to the Ce 3p_3_ core level. In the binding energy region of 930–870 eV, spin-orbit split multiplets attributed to Ce 3d are observed, representing the most distinctive spectral features of Ce [30]. The characteristic structure of Ce Auger transitions can be discerned at approximately 828.1 eV. Furthermore, signals corresponding to Ce 4p_3_ and Ce 4d are clearly identifiable in the low binding energy regions of 230–190 eV and 130–100 eV, respectively. These abundant Ce-related spectral features collectively confirm the presence and chemical environment of cerium [31]. An intense, asymmetric signal peak located at approximately 530.1 eV is assigned to O 1s, characteristic of surface oxygen species [32]. Another feature at approximately 973.1 eV corresponds to the O Auger transition. Additionally, both samples show signal peaks for C Auger, C 1s, and C 2s at approximately 1225.6, 285.6, and 18.1 eV, respectively, which are attributed to trace amounts of adventitious carbon contamination on the surface [33]. This is a common phenomenon in XPS measurements and does not affect the analysis of the chemical states of other elements.

Notably, in the inset of Figure 5, the 1 mol.% In-doped CeO_2_ sample clearly displays a pair of spin-orbit split peaks at binding energies of approximately 444.2 and 451.8 eV, corresponding to the characteristic signals of In 3d_5/2_ and In 3d_3/2_, respectively. This result directly confirms that the In element has been successfully introduced onto the material’s surface and that its chemical state is consistent with In^3+^ [34]. Combined with the absence of In-related impurity diffraction peaks and the regular contraction of lattice parameters observed in XRD analysis, it can be further confirmed that In^3+^ has been doped into the CeO_2_ lattice, rather than existing as surface-segregated species or as a separate oxide phase [35].

#### 3.3.2. O 1s and Ce 3d Analysis: V_O_ and Ce^3+^ Proportion

Figure 6a compares the O 1s core-level spectra of pure CeO_2_ and 1 mol.% In-doped CeO_2_. Both samples exhibit asymmetric broad peaks, indicating the presence of oxygen species in multiple chemical environments on the surface. Through Gaussian-Lorentzian fitting, the O 1s spectra can be deconvoluted into two characteristic peaks (Figure 6b,c). The component with a binding energy of 529.1 eV is attributed to lattice oxygen (O^2−^, i.e., oxygen in Ce-O bonds) [36]. The component located at a binding energy of 531.6 eV is generally assigned to oxygen species chemisorbed in the vicinity of *V*_O_, and its intensity is correlated with the concentration of surface *V*_O_ in the material [37].

A comparison of Figure 6a reveals that the intensity of the signal peak at 531.6 eV is significantly enhanced for the 1 mol.% In-doped CeO_2_ compared to pure CeO_2_, indicating a substantial increase in the concentration of surface *V*_O_ upon doping. To further quantify this difference, a semi-quantitative analysis was performed using the integrated area ratio of the fitted peaks. The relative concentration of *V*_O_ was defined as *A_V_*_o_/(*A*_lattice_ + *A_V_*_o_), where *A*_lattice_ and *A_V_*_o_ are the fitted areas of the lattice oxygen peak (529.1 eV) and the *V*_O_-related peak (531.6 eV), respectively [38]. The calculation results show that the relative *V*_O_ concentration for pure CeO_2_ is 29.7%, while for 1 mol.% In-doped CeO_2_, it increases significantly to 39.8%, an increase of 10.1 percentage points. This result is in excellent agreement with the higher lattice strain observed for the doped sample before calcination in XRD analysis (Table 1) and provides direct spectroscopic evidence for the charge compensation mechanism necessitated by the heterovalent substitution of Ce^4+^ by In^3+^. The increase in *V*_O_ concentration is the structural origin of the enhanced OSC observed in subsequent H_2_-TPR and the improved oxygen activation ability seen in CO-TPSR.

Figure 7a displays the Ce 3d core-level spectra of pure CeO_2_ and 1 mol.% In-doped CeO_2_. Both samples exhibit the characteristic complex multiplet splitting typical of Ce compounds. Through peak fitting (Figure 7b,c), the spectra can be deconvoluted into ten features, corresponding to the 3d_3/2_ and 3d_5/2_ spin-orbit components and their associated final state effects for Ce^4+^ and Ce^3+^ [39]. Following literature conventions, six peaks labeled *u*‴ (~916.5 eV), *u*″ (~907.2 eV), *u* (~900.6 eV), *v*‴ (~898.0 eV), *v*″ (~888.3 eV), and *v* (~881.9 eV) are assigned to Ce^4+^. Four peaks labeled *u*′ (~902.6 eV), *u*_0_ (~896.2 eV), *v*′ (~884.8 eV), and *v*_0_ (~880.1 eV) are attributed to Ce^3+^ [40,41]. Of particular note, the *v*′ peak located at approximately 884.8 eV is the most distinct signature of Ce^3+^ species. The presence of Ce^3+^ is fundamental to the OSC of CeO_2_-based materials; the oxygen storage process is essentially a reversible redox cycle between Ce^4+^ and Ce^3+^. A higher concentration of Ce^3+^ implies a greater number of reactive *V*_O_ available for participation in reactions, leading to a higher OSC [42].

To quantitatively assess the effect of In doping on the cerium valence state, the relative surface concentration of Ce^3+^ was calculated using the fitted peak area method with the formula: [Ce^3+^] = (*A*_Ce_^3+^)/(*A*_Ce_^3+^ + *A*_Ce_^4+^), where *A*_Ce_^3+^ is the sum of the areas of the four Ce^3+^ characteristic peaks, and *A*_Ce_^4+^ is the sum of the areas of the six Ce^4+^ characteristic peaks [43]. The calculation results show that the surface Ce^3+^ proportion for pure CeO_2_ is 27.6%, while for 1 mol.% In-doped CeO_2_, it increases to 30.0%. Although the magnitude of this increase (2.4 percentage points) is modest, the trend is clear and carries significant physical meaning. Combined with the substantial increase in *V*_O_ concentration observed in the O 1s analysis (29.7% to 39.8%), a complete picture of charge compensation can be constructed. Upon incorporation into the lattice substituting for Ce^4+^, the system maintains electroneutrality through two pathways: the generation of *V*_O_ (the primary pathway, corresponding to the significant enhancement of the 531.6 eV peak in O 1s), and the reduction of some Ce^4+^ to Ce^3+^ (a secondary pathway, corresponding to the modest increase in the Ce^3+^ proportion in Ce 3d). The synergistic effect of these two pathways collectively enhances the material’s *V*_O_ reservoir and redox capability [44]. These changes in surface chemical states corroborate the bulk structural features revealed by XRD, such as lattice parameter contraction and lattice strain evolution, collectively establishing the electronic structure and defect chemistry foundation for the enhanced oxygen storage performance and catalytic activity of In-doped CeO_2_ [45].

### 3.4. FTIR Analysis

**Figure 8:**
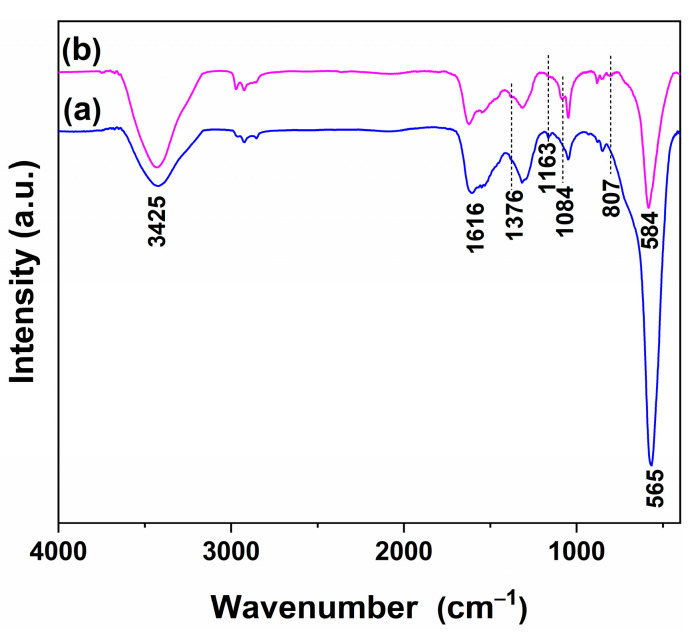
FTIR spectra of (**a**) pure CeO_2_ and (**b**) 1 mol.% In-doped CeO_2_.

To further investigate the surface chemical environments of pure and 1 mol.% In-doped CeO_2_, Fourier-transform infrared (FTIR) spectroscopy was performed, and the results are shown in Figure 8. Both samples exhibit characteristic absorption bands of CeO_2_-based materials. The broad band centered at approximately 3425 cm^−1^ is attributed to the O-H stretching vibration of surface hydroxyl groups (-OH), while the band at around 1616 cm^−1^ corresponds to the bending vibration of adsorbed molecular water [46,47]. The strong bands in the region of 560–590 cm^−1^ are assigned to the Ce-O lattice vibration [48].

Upon In^3+^ doping, several notable changes are observed. The intensity of the hydroxyl-related band at 3425 cm^−1^ increases significantly, indicating that In incorporation promotes the formation of surface –OH species. This enhancement is consistent with the increased oxygen vacancy concentration revealed by XPS (from 29.7% to 39.8%), as oxygen vacancies are known to facilitate water dissociation and hydroxyl generation on CeO_2_ surfaces [49]. Meanwhile, the Ce-O lattice vibration band shifts from 565 cm^−1^ for pure CeO_2_ to 584 cm^−1^ for the doped sample, accompanied by a noticeable decrease in intensity. The blue shift suggests a contraction of the Ce-O bond, which aligns with the lattice parameter contraction observed in XRD (from 5.4171 to 5.4129 Å) and reflects the substitution of Ce^4+^ by the smaller In^3+^ ion. The intensity decrease further implies a perturbation of the long-range ordered Ce-O lattice, consistent with the increased lattice strain and defect concentration in the doped sample. In addition, several weak but discernible new bands appear in the doped sample at 1376, 1084, and 807 cm^−1^. The bands at 1376 and 1084 cm^−1^ are typically associated with carbonate-like species or residual nitrate precursors [50], while the band at 807 cm^−1^ may be related to In-O vibrations or In-induced surface species [36]. Meanwhile, the band at 1163 cm^−1^, present in pure CeO_2_, diminishes upon doping. These spectral changes collectively indicate that In^3+^ incorporation not only modifies the local coordination environment of the Ce–O lattice but also alters the surface chemical speciation, creating a more hydroxyl-rich surface.

### 3.5. H_2_-TPR and OSC Analysis

**Figure 9:**
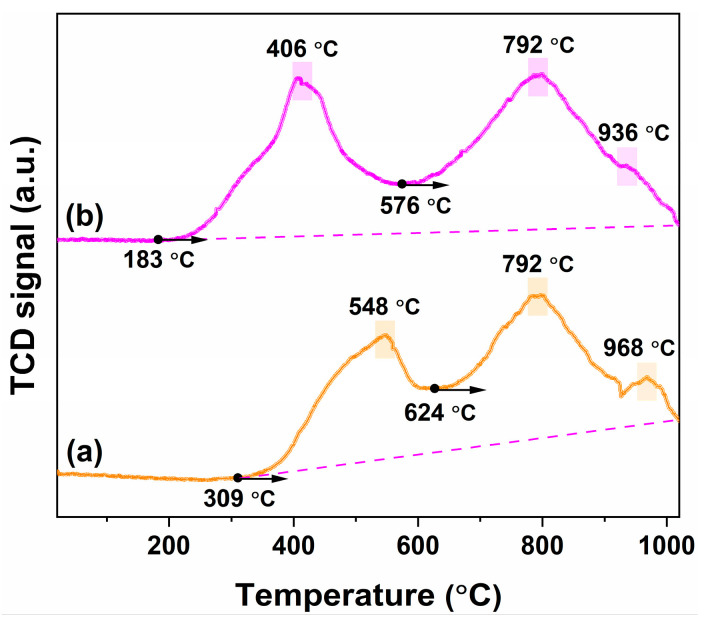
H_2_-TPR profiles of (**a**) pure and (**b**) 1 mol.% In-doped CeO_2_.

**Figure 10:**
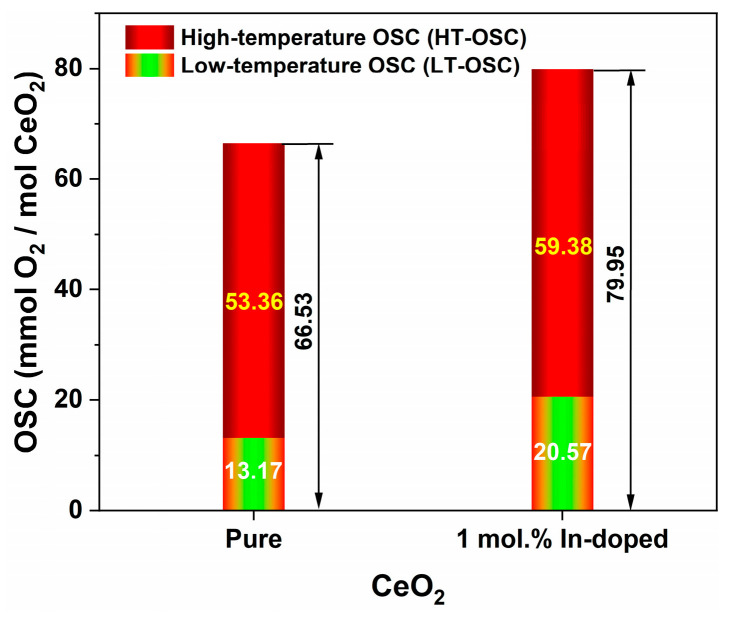
Low-temperature OSC (LT-OSC) and high-temperature OSC (HT-OSC) of pure and 1 mol.% In-doped CeO_2_. (T-OSC = LT-OSC + HT-OSC.)

Figure 9a and Figure 9b displays the H_2_-TPR profiles of pure CeO_2_ and 1 mol.% In-doped CeO_2_, respectively. The corresponding characteristic reduction temperatures and OSC derived from these profiles are summarized in Table 2, and the distribution of low-temperature and high-temperature OSC is visualized in Figure 10. Both materials exhibit typical two-stage reduction features, corresponding to the low-temperature reduction in surface or near-surface active oxygen and the high-temperature reduction in bulk lattice oxygen.

For pure CeO_2_ (Figure 9a), the onset reduction temperature (*T*_onset_) is relatively high at approximately 309 °C, indicating that its surface oxygen species are relatively stable. The subsequent low-temperature reduction peak (*LT*_peak_) appears at 548 °C, attributed to the removal of surface-adsorbed oxygen and near-surface lattice oxygen. After approximately 624 °C (*HT*_onset_), the profile enters the high-temperature reduction stage, with its main peak (*HT*_peak_) located at 792 °C, corresponding to the gradual reduction in bulk lattice oxygen. Additionally, a weak shoulder peak (*HT*_shoulder_) is observed at 968 °C, which may originate from the reduction in strongly bonded lattice oxygen. In contrast, the reduction behavior of the In-doped CeO_2_ (Figure 9b) is significantly altered. Its *T*_onset_ decreases substantially to 183 °C, indicating that In doping significantly enhances the reactivity of surface oxygen, making it more susceptible to reduction. The *LT*_peak_ correspondingly shifts to a lower temperature of 406 °C, and the peak becomes sharper, further confirming the promotional effect of doping on the kinetics of surface reduction. The *HT*_onset_ decreases from 624 °C for the pure sample to 576 °C for the doped sample, suggesting that the onset of bulk reduction occurs at a lower temperature upon doping. The *HT*_peak_ remains at 792 °C, although the transition region into this stage differs. Notably, the *HT*_shoulder_ for the doped sample appears at 936 °C, shifted to a lower temperature compared to the pure sample, and its signal is relatively weakened, suggesting that In doping partially weakens the stability of some strongly bonded lattice oxygen species.

Quantitative analysis of the reduction profiles (Table 2 and Figure 10) reveals that the low-temperature OSC (LT-OSC) increases from 13.17 mmol O_2_/mol for pure CeO_2_ to 20.57 mmol O_2_/mol for the doped sample. Similarly, the high-temperature OSC (HT-OSC) increases from 53.36 to 59.38 mmol O_2_/mol upon doping. Consequently, the total OSC (T-OSC) shows a significant enhancement, from 66.53 to 79.95 mmol O_2_/mol. These results demonstrate that In doping not only enhances the reducibility of surface oxygen species, as evidenced by the lowered *T*_onset_ and *LT*_peak_, but also substantially increases both the LT-OSC and HT-OSC, indicating an overall improvement in the material’s redox properties. Thus, In doping significantly modulates the reduction properties of CeO_2_, lowering the reduction temperature of surface oxygen while altering the high-temperature reduction characteristics, indicating an interaction between the dopant and the CeO_2_ lattice that affects the stability and reduction behavior of oxygen species at different levels. The FTIR results further reveal that In doping enriches surface hydroxyl groups (Figure 8), which are known to be reactive sites for H_2_ reduction. The increased hydroxyl coverage, together with the enhanced oxygen vacancy concentration, likely contributes to the lowered reduction onset temperature and improved low-temperature reducibility.

### 3.6. CO-TPSR for Reactant and Product Analysis

**Figure 11:**
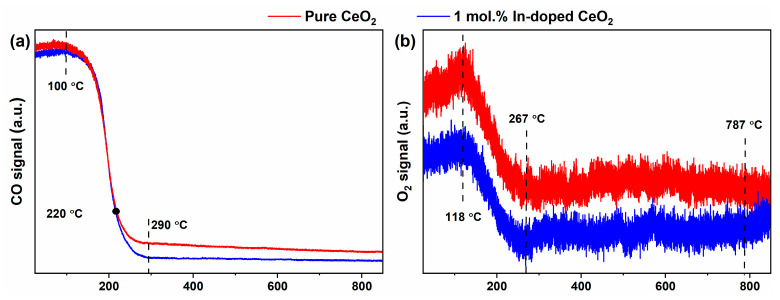
CO-TPSR spectra of pure and 1 mol.% In-doped CeO_2_ measured by on-line MS: (**a**) CO and (**b**) O_2_ signals.

**Figure 12:**
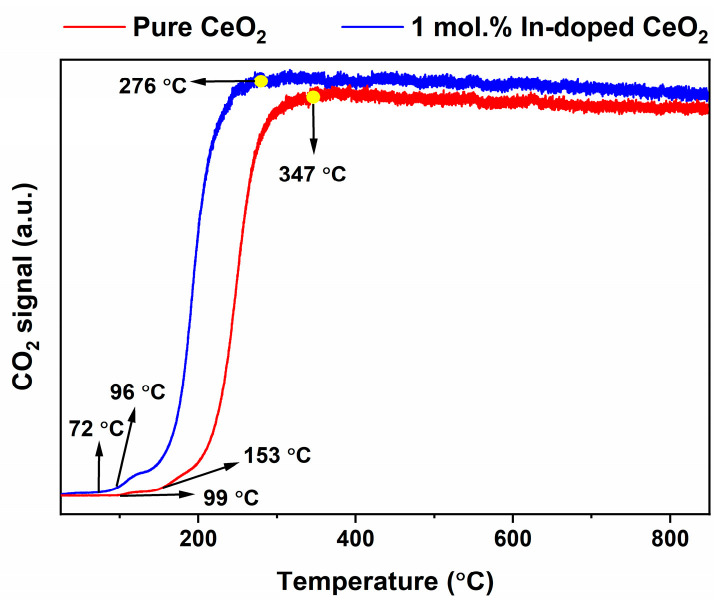
CO_2_ signal during CO-TPSR for pure and 1 mol.% In-doped CeO_2_ measured by on-line MS.

Figure 11 presents the CO-TPSR results for pure CeO_2_ and 1 mol.% In-doped CeO_2_ under a 1% CO + 10% O_2_ + 89% He atmosphere, showing the CO and O_2_ signals recorded by on-line MS. The corresponding CO_2_ generation signals are shown in Figure 12, and the characteristic temperatures derived from these profiles are summarized in Table 3. By monitoring these signals in real time, the CO oxidation activity of the two materials was systematically evaluated, and the influence mechanism of In doping on the reaction pathway and catalytic performance was elucidated.

#### 3.6.1. CO and O_2_ Signals for Reactant Consumption and Oxygen Activation

As shown in Figure 11a, in the initial stage below 100 °C, the CO signals of both samples remain stable, with the CO signal of the In-doped CeO_2_ being slightly lower than that of pure CeO_2_. This suggests that the doped sample possesses a stronger initial adsorption or oxidation capacity for CO. As the temperature increases, both CO curves exhibit a continuous downward trend, reflecting the gradual consumption of CO through oxidation. After 220 °C, the CO signal of pure CeO_2_ becomes significantly higher than that of In-doped CeO_2_, and this difference persists up to the high-temperature region. This phenomenon clearly indicates that the In-doped CeO_2_ consumes more CO over a wide temperature range, and its CO oxidation conversion rate is significantly higher than that of pure CeO_2_. After 290 °C, both curves enter a gently declining plateau, where CO consumption increases steadily without any signs of deactivation, demonstrating that both materials possess good high-temperature stability.

Figure 11b illustrates the variation of the O_2_ signal with temperature, which can be clearly divided into three stages. In the first stage from room temperature to 118 °C, the O_2_ signal shows a slight increasing trend. This is attributed to the strong OSC of CeO_2_-based materials at low temperatures, where gaseous O_2_ is stored as surface-adsorbed oxygen or lattice oxygen, leading to a relative decrease in the partial pressure of gaseous oxygen and an apparent increase in the signal. This phenomenon is a typical characteristic of the oxygen storage behavior of CeO_2_. In the second stage from 118 to 267 °C, the O_2_ signal continuously decreases, reflecting the substantial activation and continuous consumption of gaseous oxygen in the CO oxidation reaction. Compared with pure CeO_2_, the O_2_ signal of In-doped CeO_2_ is consistently lower throughout this descending interval, and its rate of decrease is faster. This indicates that the charge-compensating *V*_O_ introduced by the heterovalent substitution of In^3+^ significantly promote the adsorption and dissociation of O_2_, leading to a substantial enhancement in the material’s oxygen activation capability. In the third stage after 267 °C, the O_2_ signal enters a broad plateau region and remains essentially stable, indicating that the consumption and replenishment of gaseous oxygen have reached a dynamic equilibrium, and the CO oxidation reaction has entered a steady-state phase.

Notably, the In-doped CeO_2_ sample exhibits a slight rebound in the O_2_ signal around 787 °C. The direct implication of this phenomenon is a sudden decrease in the consumption rate of gaseous oxygen at this temperature. Possible reasons for this change include reaction pathway switching, where at high temperatures the dominant oxygen source for the CO oxidation reaction gradually shifts from gaseous O_2_ to lattice oxygen, leading to a temporary decrease in the consumption rate of gaseous oxygen [51]. Alternatively, high temperatures may induce surface reconstruction, where some *V*_O_ are filled or the number of active sites undergoes dynamic changes, temporarily reducing the efficiency of gaseous oxygen activation [52]. Another possibility is a change in *V*_O_ saturation, where the numerous *V*_O_ introduced by In doping may gradually become filled by lattice oxygen migration at high temperatures, reaching a new dynamic equilibrium and temporarily lowering the demand for gaseous oxygen [53]. Regardless of the specific mechanism, this phenomenon indirectly confirms the profound influence of In doping on the oxygen species migration ability, dynamic behavior of *V*_O_, and oxygen storage/release cycles of CeO_2_. No such rebound was observed for the pure CeO_2_ sample in this temperature range.

#### 3.6.2. CO_2_ Signal for Product Formation and Catalytic Activity Evaluation

The CO_2_ generation signal is the most direct and core indicator for evaluating CO oxidation activity. As shown in Figure 12, the CO_2_ generation behavior of the two materials differs significantly. For pure CeO_2_, the CO_2_ signal begins to rise noticeably around 99 °C (*T*_onset_), marking the onset of the catalytic reaction. It then increases sharply at 153 °C (*T*_surge_), entering a rapid oxidation phase, and forms a broad generation plateau after approximately 347 °C (*T*_plateau_). In contrast, 1 mol.% In-doped CeO_2_ exhibits superior low-temperature oxidation performance. Its *T*_onset_ is significantly advanced to 72 °C, and its *T*_surge_ is markedly shifted to 96 °C, representing decreases of 27 °C and 57 °C, respectively, compared to the pure sample. Simultaneously, *T*_plateau_ appears earlier at 276 °C, which is 71 °C lower than that of the pure sample. More importantly, the CO_2_ signal intensity of In-doped CeO_2_ is significantly higher than that of pure CeO_2_ across the entire temperature range, indicating that doping not only substantially lowers the activation energy barrier for CO oxidation but also significantly enhances the CO_2_ yield per unit time and per unit mass of catalyst. These characteristic temperatures are summarized in Table 3. The slight downward trend of the high-temperature plateau for both samples is not a sign of deactivation but rather a normal response to the reduced reactant concentration gradient and decreased mass transfer driving force. This observation is entirely consistent with the slight decrease in the CO signal plateau shown in Figure 11a, where CO consumption continues to increase slowly.

#### 3.6.3. Discussion on the Dominant Factor for Enhanced CO Oxidation

It is worth noting that the enhanced CO oxidation activity of the In-doped CeO_2_ cannot primarily be attributed to changes in morphology or specific surface area. As shown in Section 3.2, both pure and In-doped samples exhibit almost identical octahedral morphology with predominant exposure of the (111) facet (Figure 3 and HRTEM insets). The specific surface area increases only modestly from 34.4 m^2^/g to 40.2 m^2^/g upon doping (Table 1). Moreover, in our previous studies on doped CeO_2_ systems, we have demonstrated that specific surface area is not the dominant factor governing the oxygen storage capacity (OSC) or catalytic performance [54]. In some cases, even a decrease in surface area was observed after doping, while the OSC still increased significantly [55]. Therefore, the improved CO oxidation performance, as evidenced by the decreased light-off temperature from 99 °C to 72 °C and the lowered rapid oxidation temperature from 153 °C to 96 °C, should be attributed primarily to the enhanced defect chemistry induced by In^3+^ doping, namely the increased oxygen vacancy concentration from 29.7% to 39.8% and the elevated Ce^3+^ fraction from 27.6% to 30.0%, rather than the modest changes in morphology or surface area.

### 3.7. By-Product Signals and Mechanistic Insights

**Figure 13:**
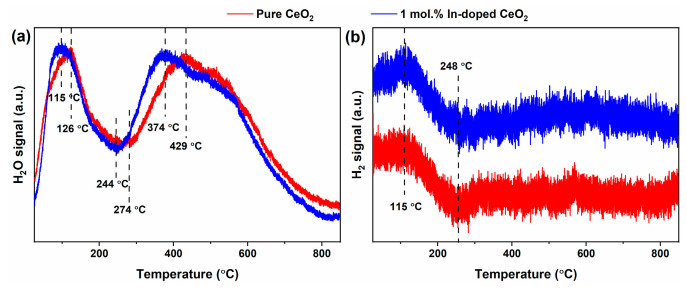
By-product signals detected by MS during CO-TPSR: (**a**) H_2_O and (**b**) H_2_ of pure and 1 mol.% In-doped CeO_2_.

During CO-TPSR, distinct H_2_O and H_2_ signals were detected alongside CO_2_, as shown in Figure 13a,b. These by-products provide key evidence for the participation of surface hydroxyl groups in CO oxidation. As shown in Figure 13a, the H_2_O signals for both samples exhibit a clear double-peak feature. For pure CeO_2_, the two peaks are located at 126 and 429 °C. For In-doped CeO_2_, both shift to lower temperatures (115 and 374 °C), with reductions of 11 and 55 °C, respectively. This trend is consistent with the decreased CO_2_ onset temperatures (Figure 12), indicating that In doping promotes hydroxyl (-OH) participation in CO oxidation. The slightly higher H_2_O intensity for the doped sample suggests that -OH groups are actively consumed by CO rather than passively desorbed. The low-temperature peak is attributed to terminal hydroxyls (≡Ce-OH) reacting with CO, while the high-temperature peak corresponds to bridging or near-bulk hydroxyls. The downward shift in both peaks demonstrates that In doping lowers the reaction energy barrier for hydroxyl-mediated oxidation.

Figure 13b displays clear H_2_ signals with a distinct peak-shaped feature. Both samples show a maximum around 115 °C, followed by a gradual decrease to a low-intensity plateau after 248 °C. The H_2_ signal for In-doped CeO_2_ is significantly higher across the entire temperature range. H_2_ generation arises from recombinative desorption of surface hydrogen atoms (2H* → H_2_), with H* originating from the reaction CO + OH → CO_2_ + H*, a key step in the water-gas shift reaction. The mirror-image relationship between H_2_ and O_2_ signals (Figure 11b) reflects dynamic competition between H* generation and oxidation. The enhanced H_2_ signal upon doping directly demonstrates accelerated kinetics of the hydroxyl-mediated CO oxidation pathway, consistent with the increased H_2_O intensity, lowered CO_2_ onset, and enhanced O_2_ consumption.

The by-product signals are critical for understanding the reaction mechanism. The FTIR analysis provides direct structural evidence for the enhanced surface hydroxylation upon In doping (Figure 8), which is consistent with the observed H_2_O and H_2_ evolution during CO-TPSR. Specifically, the increased –OH coverage offers abundant reactive sites for the hydroxyl-mediated CO oxidation pathway. The shift of H_2_O peaks to lower temperatures confirms that In doping reduces the energy barrier for -OH participation. The enhanced H_2_ signals provide direct evidence for the CO + OH → CO_2_ + H step. Together with the main reaction data, these results reveal that In doping synergistically activates both gas-phase oxygen and hydroxyl-mediated pathways, providing multi-angle support for the enhanced CO oxidation performance.

## 4. Conclusions

Saturated In^3+^ doping at 1 mol.% significantly enhances the *V*_O_ concentration and surface Ce^3+^ fraction of nano-octahedral CeO_2_ through heterovalent substitution, resulting in a contracted lattice parameter from 5.4171 to 5.4129 Å. This defect chemistry modification greatly improves the reducibility of surface oxygen species, lowering the reduction peak temperature from 548 to 406 °C and the onset reduction temperature from 309 to 183 °C. Consequently, the low-temperature OSC increases from 13.17 to 20.57 mmol O_2_/mol and the high-temperature OSC from 53.36 to 59.38 mmol O_2_/mol. For CO oxidation, the doped sample shows a decreased light-off temperature from 99 to 72 °C and a lowered rapid oxidation temperature from 153 to 96 °C. Notably, the detection of H_2_O and H_2_ signals during CO-TPSR, together with FTIR evidence of enriched surface hydroxyl groups, suggests the involvement of a hydroxyl-mediated reaction pathway. Overall, saturated In^3+^ doping provides an effective strategy for enhancing the low-temperature redox and catalytic performance of nano-octahedral CeO_2_.

## Figures and Tables

**Table 1:** Structural parameters and *S*_BET_ of pure CeO_2_ and 1 mol.% In-doped CeO_2_ before and after calcination in air at 500 °C for 1 h.

Sample	Crystallite Size (nm)	Relative Crystallinity (%)	Strain (%)	Lattice Parameters (Å)	*S*_BET_ (m^2^/g)
Pure CeO_2_ (Before calcination)	18.2	63.2	0.481	5.4278	/
Pure CeO_2_ (After calcination)	27.4	64.3	0.361	5.4171	34.4
1 mol.% In-doped CeO_2_ (Before calcination)	17.8	61.2	0.540	5.4205	/
1 mol.% In-doped CeO_2_ (After calcination)	30.2	62.3	0.349	5.4129	40.2

**Table 2:** Characteristic reduction temperatures and OSC of pure and 1 mol.% In-doped CeO_2_ derived from H_2_-TPR.

Sample	Reduction Temperatures (°C)					OSC (mmol O_2_/mol)		
	*T*_onset_	*LT*_peak_	*HT*_onset_	*HT*_peak_	*HT*_shoulder_	LT-OSC	HT-OSC	T-OSC
**Pure CeO_2_**	309	548	624	792	968	13.17	53.36	66.53
**1 mol.% In-doped CeO_2_**	183	406	576	792	936	20.57	59.38	79.95

**Table 3:** Characteristic CO_2_ generation temperatures of pure and 1 mol.% In-doped CeO_2_ during CO-TPSR.

Sample	*T*_onset_ (°C)	*T*_surge_ (°C)	*T*_plateau_ (°C)
**Pure CeO_2_**	99	153	347
**1 mol.% In-doped CeO_2_**	72	96	276

## Data Availability

Data are contained within the article.
